# Desvenlafaxine May Accelerate Neuronal Maturation in the Dentate Gyri of Adult Male Rats

**DOI:** 10.1371/journal.pone.0098530

**Published:** 2014-06-04

**Authors:** Aditya Asokan, Alan R. Ball, Christina D. Laird, Linda Hermer, Brandi K. Ormerod

**Affiliations:** 1 J. Crayton Pruitt Family Department of Biomedical Engineering and Evelyn F. & William L. McKnight Brain Institute, University of Florida, Gainesville, Florida, United States of America; 2 Department of Psychology and Behavioral Neuroscience Program, University of Florida, Gainesville, Florida, United States of America; University of South Florida, United States of America

## Abstract

Adult hippocampal neurogenesis has been linked to the effects of anti-depressant drugs on behavior in rodent models of depression. To explore this link further, we tested whether the serotonin-norepinephrine reuptake inhibitor (SNRI) venlafaxine impacted adult hippocampal neurogenesis differently than its primary active SNRI metabolite desvenlafaxine. Adult male Long Evans rats (n = 5–6 per group) were fed vehicle, venlafaxine (0.5 or 5 mg) or desvenlafaxine (0.5 or 5 mg) twice daily for 16 days. Beginning the third day of drug treatment, the rats were given a daily bromodeoxyuridine (BrdU; 50 mg/kg) injection for 5 days to label dividing cells and then perfused 2 weeks after the first BrdU injection to confirm total new hippocampal cell numbers and their phenotypes. The high desvenlafaxine dose increased total new BrdU^+^ cell number and appeared to accelerate neuronal maturation because fewer BrdU^+^ cells expressed maturing neuronal phenotypes and more expressed mature neuronal phenotypes in the dentate gyri of these versus vehicle-treated rats. While net neurogenesis was not increased in the dentate gyri of rats treated with the high desvenlafaxine dose, significantly more mature neurons were detected. Our data expand the body of literature showing that antidepressants impact adult neurogenesis by stimulating NPC proliferation and perhaps the survival of neuronal progeny and by showing that a high dose of the SNRI antidepressant desvenlafaxine, but neither a high nor low venlafaxine dose, may also accelerate neuronal maturation in the adult rat hippocampus. These data support the hypothesis that hippocampal neurogenesis may indeed serve as a biomarker of depression and the effects of antidepressant treatment, and may be informative for developing novel fast-acting antidepressant strategies.

## Introduction

Despite the prevalence of typically recurrent depressive disorders worldwide, their etiologies and pathophysiologies remain relatively enigmatic [1], [2]. The discoveries that thousands of neurons are added to olfactory bulbs and hippocampal dentate gyri of mammals including humans each day throughout life [3]–[10] stimulated research geared toward understanding the role of adult neurogenesis in normal cognition and dysregulated adult neurogenesis in cognitive decline and mental health disorders. The discoveries that antidepressants potentiate the proliferation of neural progenitor cells (NPCs) and the survival of their neuronal progeny [11]–[15] generated excitement in the research community that a novel mechanism and therapeutic target for antidepressant strategies may have been identified.

Links between adult hippocampal neurogenesis and depression were drawn in early studies investigating how adult neurogenesis is regulated. Chronic exposures or responses to stressors are hypothesized to increase depression risk [16] and chronic exposures to stressors or stress-level hypothalamic-pituitary-adrenal axis (HPA) hormones decreases NPC division [17], [18]. Dysregulated serotonin transmission has long been implicated in depression [1] and the deleterious effects of serotonin depletion on dividing subventricular and subgranular zone NPCs can be reversed by grafted fetal raphe neurons that secrete supranormal serotonin levels constitutively [19]–[21]. Several weeks of treatment with selective serotonin reuptake inhibitors (SSRIs), norephinephrine selective reuptake inhibitors (NRIs), tricyclic antidepressants (TCA), monoamine oxidase inhibitors (MAOI) or triple monoamine reuptake inhibitors can alleviate the symptoms of depression and stimulate NPC division and the survival of their neuronal progeny [12], [22]–[24]. Although the link between hippocampal neurogenesis, depression and antidepressants is more difficult to study in humans, some evidence derived from post-mortem tissue samples suggests antidepressant treatment even stimulates NPC division in human patients [25], [26]. An emerging theory is that antidepressants may restore hippocampal neurogenesis and therefore the ability to discriminate contexts, which treats the anxiety disorder by reducing the tendency to overgeneralize [27].

The hypothesis that antidepressant drugs may mediate some therapeutic effects by stimulating adult neurogenesis exhibits face validity because their effects are typically observed after several weeks of use and new hippocampal neurons mature morphologically and functionally over several weeks [4], [24], [28]. A handful out of several studies conducted have shown that ablating both hippocampal and olfactory bulb neurogenesis can increase the incidence of behaviors interpreted as anxiety- or depression-related in animal models and may, in fact, render susceptibility in animal models [29], [30]. Perhaps more compelling evidence suggests that chronic antidepressant treatment requires neurogenesis to alleviate the behavioral symptoms in animal models of anxiety and depression [13], [31]–[33]. Taken together, these studies suggest that research elucidating the role that new neurons play in mood and in the effects of antidepressants on mood disorders could promote the development of novel treatment strategies.

This study tested the effects of the serotonin-norepinephrine reuptake inhibitor (SNRI) antidepressants venlafaxine and desvenlafaxine succinate on adult hippocampal neurogenesis. Venlafaxine is converted in the liver to its active metabolite *O*-desmethylvenlafaxine (the free base of desvenlafaxine succinate) by the cytochrome P450 enzyme CYP2D6 [34]. Both venlafaxine and desvenlafaxine succinate are now marketed as SNRI antidepressants that competitively bind serotonin (5-HT) transporters with relatively similar affinities (Ki = 82 nM and 40.2 nM, respectively) and at higher doses norepinephrine (NE) transporters with variable affinities (Ki = 2480 nM and 558.4 nM, respectively), possibly with weak dopamine (DA) transporter binding [35]–[37]. Theoretically these SNRIs should produce similar effects but the pharmacokinetic properties of desvenlafaxine and some clinical data suggest that desvenlafaxine may produce a faster response onset [38]–[40]. Desvenlafaxine succinate is active without metabolic catalysis and exhibits low plasma-binding properties which produces linear and dose-proportional pharmacokinetics and steady state plasma concentrations within days making the initial dose the effective dose [39], [40]. Clinical desvenlafaxine succinate doses generally produce ∼2× higher serum *O*-desmethylvenlafaxine concentrations than clinical venlafaxine doses within a similar time frame [39], [40]. Finally, venlafaxine exhibits a shorter half-life (∼5 versus 11 h) faster clearance rates, lower bioavailability and lower drug accumulation systemically and in the brain than desvenlafaxine [39], [41]. A two week venlafaxine course has been shown previously to potentiate hippocampal NPC proliferation and to enhance the survival of new cells [11], [14]. Based upon the pharmacokinetics of desvenlafaxine and venlafaxine, we hypothesized that desvenlafaxine may impact adult hippocampal neurogenesis differently than venlafaxine.

## Material and Methods

### 2.1 Subjects

All rats used as subjects in this study were treated in accordance with the policies set forth by the University of Florida Institutional Animal Care and Use Committee and the National Institutes of Health Guide for the Care and Use of Laboratory Animals (NIH Publications No. 80–23) regarding the ethical use of animals for experimentation. The University of Florida Institutional Animal Care and Use Committee approved this study. Male Long Evans rats (8 weeks-old; n = 26) were housed singly upon arrival from Charles River (Wilmington, MA) in cedar bedding-lined shoebox cages located in a specific pathogen-free colony room maintained on a 12∶12 hour light∶dark cycle (lights on at 7:00am) at 24±1°C. The rats were given free access to Harlan Teklad irradiated rat chow and water filtered by reverse osmosis for the duration of the experiment.

The rats were habituated to the colony room for a week after arrival and then handled and weighed every other day for 2 weeks. At the beginning of the 3^rd^ week after arrival, the rats were fed Nestle strawberry milk vehicle (500 µl) once per day in their cages for 3 days and then Nestle strawberry milk (500 µl) in Jiffy peanut butter vehicle (0.6 g; see Antidepressant drug preparation and administration section for details) or antidepressants (0.5 or 5 mg) twice per day (12 h apart) for 16 days. The rats were weighed daily over the 16 days of drug treatment. Beginning the 3^rd^ day of anti-depressant treatment, the rats were given a single intraperitoneal (i.p.) injection of the cell synthesis marker bromodeoxyuridine (BrdU; 50 mg/kg) once per day over 5 days to label dividing cells. Two weeks after the initial BrdU injection, the rats were perfused to quantify new BrdU^+^ hippocampal cell numbers stereologically and their phenotypes under confocal microscopy.

### 2.2 Antidepressant drug preparation and administration

Effexor XR (venlafaxine; Pfizer Inc., Mission, KS) 150 mg tablets and Pristiq Extended-Release (desvenlafaxine succinate; Pfizer Inc., Mission, KS) 50 mg tablets were crushed with a mortar and pestle and then suspended in Nestle strawberry milk at a concentration of either 1 mg/ml or 10 mg/ml. Strawberry milk vehicle and strawberry milk containing each drug dose was frozen at 20°C in 500 µl aliquots to prevent spillage when presented to the rats. Although the rats readily consumed frozen strawberry milk vehicle over 3 days before drug treatment, they refused to consume strawberry milk containing antidepressant, potentially because of the bitter taste detected by one of the experimenters. We therefore added 0.6 g of Jiffy peanut butter to each treat to mask the bitterness and visually confirmed that each singly housed rat readily consumed its vehicle or drug-supplemented treat twice daily (at 10a.m. and at 10p.m.) for the duration of the 16 day-long experiment. Because the rats weighed 436.67±6.01 g on the first day of drug treatment and 489.57±7.33 g before perfusion, the daily dose of each antidepressant drug was 2.0–2.3 mg/kg or 20.0 mg–23.0 mg/kg per day. The low dose falls within the range of effective clinical doses for both drugs (Effexor XR: 75–375 mg/day or ∼0.7–5.4 mg/kg and Pristiq ER: 50–400 mg/day or ∼0.7–5.7 mg/kg) and the high dose, employed in several animal studies, is about 4× the current maximum recommended clinical venlafaxine dose (350 mg/day or ∼5.4 mg/kg) and about twice the current recommended maximum desvenlafaxine succinate dose (750 mg/day or ∼10 mg/kg; Deecher et al., 2006; Lourenco and Kennedy, 2009; Preskorn, 2009).

### 2.3 Bromodeoxyuridine preparation and injection

BrdU (Sigma Aldrich, St. Louis, MO) was dissolved at a concentration of 20 mg/ml in fresh isotonic sterile saline prepared just prior to use and the BrdU solution was injected in a volume of 2.5 ml/kg once per day over 5 days. We have confirmed expected age- and inflammation-associated group differences in hippocampal neurogenesis in the adult rat and mouse using multiple injections of this 50 mg/kg BrdU dose [42], , which does not appear to induce cell death [5] or impact adult rodent health negatively [44].

### 2.4 Perfusion and histology

The day after the final antidepressant drug treatment, the rats were anaesthetized with an i.p. injection of ketamine (100 mg/ml) and xylazine (10 mg/ml) dissolved in isotonic saline and were then perfused transcardially with ice cold saline and then freshly prepared ice cold 4% paraformaldehyde (Electron Microscopy Sciences, Hatfield, PA). Brains were extracted and stored overnight in perfusate before being equilibrated in a 30% sucrose solution at 4°C. The brains were sectioned coronally at 40 µm intervals through their rostral-caudal extent on a freezing stage microtome (Model 860; American Optical Corporation; IMEB Inc., San Marcos, CA) and the sections stored in cryoprotectant solution (30% ethylene glycol, 25% glycerol and 45% 0.1 M sodium phosphate buffer) at −20°C until stained immunohistochemically.

### 2.5 Immunohistochemistry

New BrdU^+^ cells were revealed on one 1-in-12 series' of systematically uniform sections (spaced 480 µm apart) taken through the rostral-caudal extent of the dentate gyrus using standard 3, 3-diaminobenzidine (DAB, Sigma, St. Louis, MO) peroxidase stain and an ABC labeling kit (Vector Laboratories, Burlingame, CA) so that total numbers could be estimated using stereological principles [43], [45]–[47]. We randomly selected which of the 12 collected sets of sections to process immunohistochemically for each rat to ensure that the first section in each rat's set was randomly the 1^st^–12^th^ section, which contained at least the medial/infrapyramidal blade of the dentate gyrus with visible granule cell layer (between ∼−1.92 and −2.40 mm posterior to bregma) according to Paxinos and Watson (Paxinos and Watson, 2007). The sections were incubated in 0.6% H_2_O_2_ for 10 minutes to quench endogenous peroxidase, rinsed repeatedly in 0.9% saline and then incubated in 2N HCl at 37°C for 20 min to denature DNA. After a 20 min incubation in blocking solution (3% normal donkey serum +0.3% Triton-X in TBS) at RT, the sections were incubated overnight in rat anti-BrdU (1∶500 in blocking solution; Accurate Chemical, Westbury, NY; OBT0030-CX) at 4°C. The following day, tissue was incubated in biotinylated SP anti-rat IgG (1∶500 in blocking solution; Jackson Immunoresearch; West Grove, PA) solution for 4 h at room before being complexed with avidin-biotin horseradish peroxidase solution (PK 6100; Vector Labs, Burlingame, CA) for 20 min and then developed in a solution of tris-buffered saline (TBS; pH 7.4) containing 0.02% diaminobenzene tetrahydrochloride (DAB) and 0.5% H_2_O_2_ for 2–5 min. Sections were rinsed repeatedly in TBS between steps and after processing, were mounted on glass microscope slides, dried overnight, dehydrated in an alcohol series and then coverslipped under permount.

The phenotypes of BrdU^+^ cells were confirmed under confocal microscopy on 3–4 randomly selected sections per rat that were stained using fluorophore-conjugated secondary antibodies (1∶500; Jackson Immunoresearch, West Grove, PA). These sections were rinsed repeatedly in TBS between steps and all antibodies were diluted in blocking solution. The sections were incubated in blocking solution for 20 min and then overnight in either: 1) the mature neuronal marker mouse anti-neuronal nucleii (NeuN; 1∶500; Chemicon, Temecula, CA) and the immature neuronal marker goat anti-doublecortin (DCX; 1∶500; Santa Cruz Biotechnology, Santa Cruz, CA), 2) the astrocyte marker chicken anti-glial fibrillary acidic protein (GFAP; 1∶1000; EnCor Biotechnology, Alachua, FL) or 3) the oligodendrocyte precursor marker rabbit anti-chondroitin sulfate proteoglycan (NG2; 1∶500; Chemicon, Temecula, CA). The following day, the sections were incubated for 4 h at RT in either: 1) anti-mouse IgG FITC and anti-goat IgG Cy5, 2) anti-chicken IgG Cy5 or 3) anti-rabbit IgG FITC and then fixed for 10 min in 4% para-formaldehyde. After several 0.9% saline solution rinses, the sections were incubated in 2N HCl solution for 20 min at 37°C and then incubated overnight in rat anti-BrdU, anti-rat IgG Cy3 for 4 h at RT and then 4′,6-diamidino-2-phenylindole (DAPI; 1∶10000; Chemicon, Temeculah, CA) for 10 min to label all nuclei before being rinsed and mounted under PVA DABCO (2.5% DABCO, 10% poly vinyl alcohol and 20% glycerol in TBS).

### 2.6 Data Analysis

#### Stereological estimates of new cell numbers

The total number of BrdU^+^ cell numbers in the subgranular zone (SGZ; the 2–3 cell diameter distance between the granule cell layer and hilus) and granule cell layer was estimated using sections on which BrdU^+^ cells was revealed using peroxidase immunohistochemistry. Because BrdU^+^ cells are located irregularly, BrdU^+^ cells were counted exhaustively on every 1-in-12 series of sections (8 sections per rat) under a 40× objective on a Zeiss Axio observer Z1 inverted microscope (Carl Zeiss, Thornwood, NY). To generate the stereological estimate, the total number of BrdU^+^ cells counted was multiplied by the section interval of 12. SGZ and granule cell areas on which new cells were counted was measured using Axiovision software (Carl Zeiss, Thornwood, NY) and BrdU^+^ cell densities were calculated by dividing the total BrdU^+^ cell numbers by the total area they were counted upon.

#### Analysis of new cell phenotypes

The phenotype of at least 50 BrdU^+^ cells per rat in each staining set was confirmed under a 40× objective (with 2.3× digital zoom) on a Zeiss LSM 710 laser scanning confocal microscope with 405 (used to excite DAPI) 488 (used to excite FITC), 510, 543 (used to excite Cy3) and 633 (used to excite Cy5) nm laser lines. Laser intensities were always maintained below 10%. New cells were scanned through the z-plane and the percentage of BrdU/DAPI-labeled nuclei associated unambiguously with DCX and/or NeuN, GFAP, or NG2 or unassociated with the phenotypic markers employed (unlabeled) were calculated. The number of new cells of each phenotype was calculated by multiplying the percentage of each phenotype observed with the total number of BrdU^+^ cells estimated.

#### Morphological analysis of the maturation state of new immature neurons

The maturity of at least 50 BrdU/DCX^+^ neurons distributed throughout the infra- and supra-pyramidal blades of granule cell layer per rat was examined by categorizing them based upon the degree and extent of dendritic branching and by measuring soma perimeters. Confocal images were taken through the somatodendritic extent of BrdU/DCX^+^ cells that appeared to have dendritic branches contained entirely within the section being scanned. The upper and lower extent of the z-plane containing the cell of interest was set using Zen software and images were taken at 0.45 µm steps through the entire z-plane using a 40× objective on the Zeiss LSM 710 scanning confocal microscope described previously. The images were projected using the Zen 2009 software (Carl Zeiss, Thornwood, NY) maximum projection function. The BrdU/DCX^+^ cells were then grouped into one of 6 categories of maturation based upon the length and degree of branching among dendrites as described by Plumpe and colleagues (2006) who argue that cells with the shortest and least branched dendrites are likely early post-mitotic cells while those with longer more extensively branched dendrites are likely more mature post-mitotic cells. In the current study we defined 1) cells with no processes as Category A cells, 2) cells with unbranched processes that did not penetrate the granule cell layer as Category B cells, 3) cells with un-branched processes that penetrated the granule cell layer as Category C cells, 4) cells with un-branched processes that penetrated the molecular layer as Category D cells, 5) cells with sparsely branched processes that penetrated the molecular layer Category E cells and 6) cells with elaborately branched processes that penetrated the molecular layer Category F cells. The percentage of all BrdU/DCX^+^ cells in each category was calculated.

For a more elaborate analysis of BrdU/DCX^+^ cell morphologies, z-stack images of 10 randomly selected cells (the z-plane contained the somatodendritic extent of each cell) from each rat were projected through their z-plane into a single maximum intensity 2D image in which the cell body and any dendritic branches could be visualized clearly. Cell body area and perimeter, dendritic branch number, total branch length (i.e. the sum total of the individual branch length) and dendritic branch node was quantified using the Zen 2009 software open and closed Bezier functions. All measurements are expressed in µm or µm^2^.

### 2.7 Statistical Analysis

All statistical analyses were conducted using Statistica software version 10 (Statsoft, Tulsa, OK). The effect of drug treatment (vehicle, VEN-LO, VEN-HI, DES-LO and DES-HI) on the dependent variables (total new cell number, total new neuron, new astrocyte, or new oligodendrocyte number, and immature neuron soma size, dendritic branch number and length) was tested using analyses of variance (ANOVAs). Significant effects were explored using Duncan's post hoc range tests. The alpha level for all statistical tests was set at *p*≤0.05.

## Results

### 3.1 Rats exhibited good general health for the duration of the experiment

**Figure 1:**
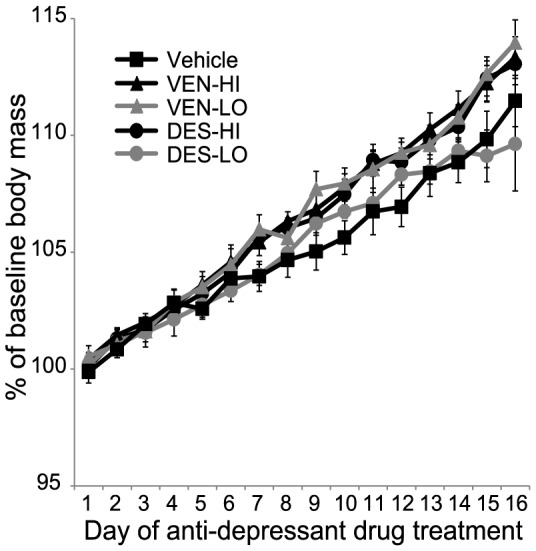
Anti-depressants increased body weight gain in the second week of treatment. Daily weights taken throughout the 16 days of antidepressant treatment confirmed normal growth among vehicle (n = 5) and DES-LO (n = 5) treatment groups and reveal that the low (n = 5) and high (n = 6) doses of venlafaxine or the high desvenlafaxine dose (n = 5) promoted weight gain in male rats beyond that expected of normal growth. Specifically, greater % changes from baseline body masses were detected during the second week of antidepressant drug treatment in VEN-HI rats (*p values*<0.05 for Days 10, 12, 14 and 15), VEN-LO rats (*p values*<0.05 for Days 9, 10, 12, 15 and 16) and DES-HI rats (*p values*<0.05 for Days 10, 11, 12, 15 and 16) relative to Vehicle-treated rats. Data are expressed as mean ± S.E.M.

Daily visual inspection of the rats confirmed healthy appearances with no overt change in posturing, grooming or porphyrin secretion over the duration of drug treatment. Figure 1 shows that the percentage of baseline body mass calculated on each day during drug treatment changed across days (*F*_(15, 315)_ = 399.4; p<0.001) and that the daily change interacted significantly with antidepressant drug treatment (*F*_(60, 315)_ = 2.2; *p*<0.001). Body mass was consistent across groups on the first day of drug treatment (*p values*>0.80). As expected of young male rats, body mass increased relative to baseline on each day of drug treatment (*p values*<0.05) with the exception of Day 12 on which body mass only tended to increase relative to Day 11 (0.10>p>0.05). In the second week, body mass increases were significantly greater in VEN-HI rats (*p values*<0.05 for Days 10, 12, 14 and 15), VEN-LO rats (*p values*<0.05 for Days 9, 10, 12, 15 and 16) and in DES-HI rats (*p values*<0.05 for Days 10, 11, 12, 15 and 16) relative to vehicle-treated rats. These data suggest that twice daily treatment with either dose of venlafaxine or the high desvenlafaxine dose for about a week promotes weight gain in young adult male rats beyond normal growth.

### 3.2 Desvenlafaxine treatment increased total new cell number

**Figure 2:**
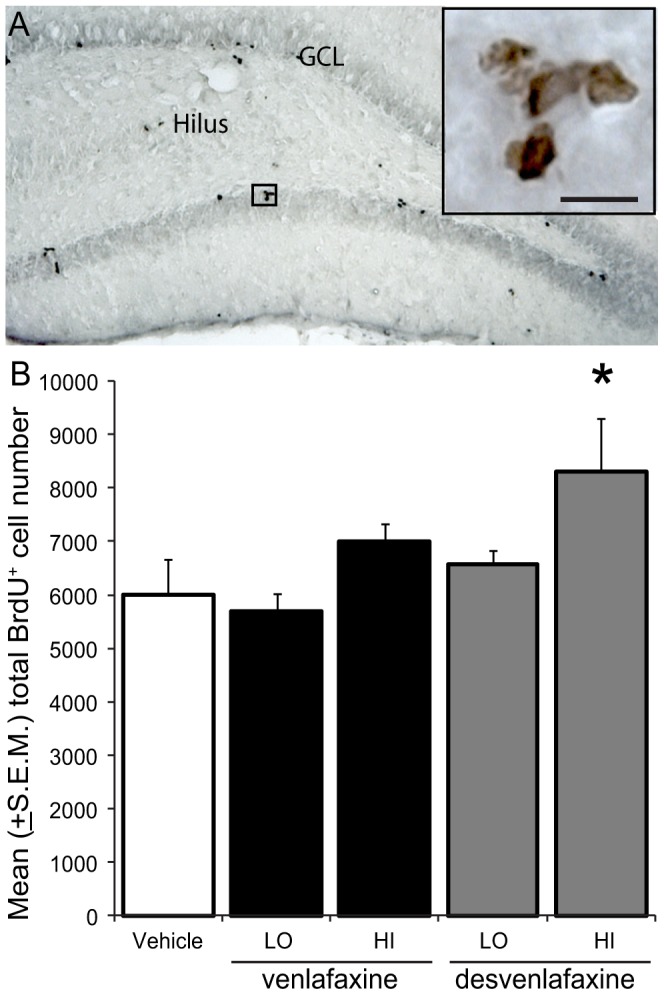
High dose desvenlafaxine increases NPCproliferation and survival in the DG of rats. **A**)Representative brightfield image of BrdU^+^ cellsrevealed in the dentate gyrus of a vehicle-treated rat using DAB BrdU (inbrown). Scale bar  = 50 µm. B) Stereological estimates revealed more 10–14day-old BrdU^+^ cells in the dentate gyriof DES-HI- versus vehicle-treated rats (*p* = 0.01*).

Figure 2A shows a representative example of BrdU^+^ cells counted in the SGZs and GCLs of rats in each treatment group and Figure 2B shows the stereologically estimated mean (±S.E.M.) total number of BrdU^+^ cells found in these regions. Anti-depressant drug treatment significantly affected total BrdU^+^ cell number in the dentate gyri of adult male rats (*F*_(4,21)_ = 3.16; *p*<0.05). Specifically, more new cells were found in the dentate gyri of rats treated with high dose of desvenlafaxine versus vehicle (*p*<0.05). Since 2 weeks elapsed between the first BrdU injection and perfusion, the effect of high desvenlafaxine on total new cell number could reflect stimulated NPC proliferation and/or enhanced new cell survival.

### 3.3 Desvenlafaxine may accelerate neuronal maturation in the dentate gyri of adult male rats

**Figure 3:**
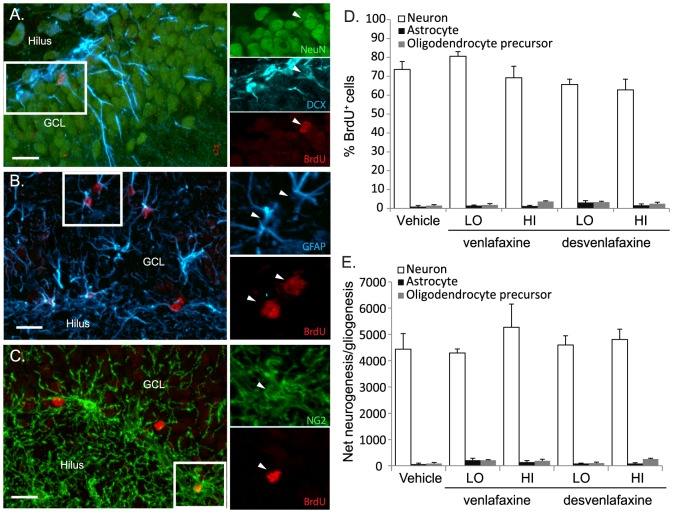
Anti-depressant treatment doesnot influence NPC differentiation in the DG of adult rats. A) Representative confocal images of a new transitionstate neuron (**A**), astrocytes (B) and an oligodendrocyte precursor (**C**)in the dentate gyri of adult male rats. Figure (**A**)shows new BrdU^+^ cells in red, DCX^+^immature neurons in cyan and NeuN^+^ mature neuronsin green. The inset shows a new transition state neuron thatwas BrdU^+^ (red) and expressed bothDCX (Cyan) and NeuN (green). Each signalis shown separately in the panels. Figure **B**)shows new BrdU^+^ cells in red and astrocytes ingreen. The inset shows new astrocytes that were BrdU^+^and expressed GFAP. Each signal is shown separately in the panels.Figure **C**) shows new BrdU^+^cells in red and NG2^+^ oligodendrocytes precursorsin green. The inset shows a new NG2^+^oligodendrocyte precursor (in green) that was BrdU^+^ (red).Each signal is shown separately in the panels. **D**)Although a greater % of new (BrdU^+^)cells acquired a neuronal versus astrocyte or oligodendrocyte precursor phenotype,phenotype was not impacted by antidepressant treatment. E) Similarly, total new neuron number was greaterthan either the total new astrocytes or new oligodendrocytes precursor number (p values <0.0001) but the total number of newneurons and glia was unaffected by antidepressant treatment (*p* = 0.84).All data are expressed as mean ± S.E.M.

Figure 3 shows confocal images of representative sections of the dentate gyrus stained immunohistochemically to reveal new BrdU^+^ cells (in red) expressing immature DCX^+^ (in cyan), transitioning DCX/NeuN^+^ (in cyan and green) or mature NeuN^+^ (in green) neuronal phenotypes (Figure 3A), GFAP^+^ astrocyte phenotypes (in cyan; found in the dentate gyri of all rats (Figure 3B) and NG2^+^ oligodendrocytes precursors phenotypes (in green; Figure 3C). Figure 3D shows the % of BrdU^+^ cells expressing each phenotype and Figure 3E shows the total number of new neurons, astrocytes and oligodendrocytes (the total number of new cells in Figure 2 multiplied by the % of each phenotype in Figure 3D). Similar percentages of BrdU^+^ cells expressed neuronal (*F*_(4,21)_ = 2.13; *p*>0.05), astrocyte (*F*_(4,21)_ = 1.53; *p*>0.05) or oligodendrocyte precursor (*F*_(4,21)_ = 2.18; *p* = 0.05) phenotypes (Figure 3D and Table 1 ‘Neuronal Column’) across antidepressant treatment groups. With respect to net neurogenesis and net gliogenesis, significantly more new neurons were detected than either new oligodendroctye precursors or new astrocytes (*p values*<0.0001) in all rats combined (effect of phenotype: *F*_(2, 42)_ = 371.89; *p*<0.0001) but neither new neuron, new astrocyte nor new oligodendrocyte precursor number varied across antidepressant treatment groups (Figure 3E; effect of group: *F*_(4, 21)_ = 0.56; *p*>0.05 and interaction effect: *F*_(8, 42)_ = 0.52; *p*>0.05).

**Table 1:** Antidepressant Treatment Impacts The % BrdU^+^ Cells Expressing Neuronal Markers.

Group	Neuronal	DCX^+^	DCX/NeuN^+^	NeuN^+^
**Vehicle**	73.6±4.2	20.6±3.4	33.1±2.0	19.9±3.2
**Ven-Lo**	80.6±2.4	30.2±3.1	37.1±2.5	13.2±1.2
**Ven-Hi**	69.2±6.1	26.7±4.4	23.8±3.4	18.7±7.3
**Des-Lo**	65.7±2.8	14.3±2.1	31.6±3.1	19.9±3.6
**Des-Hi**	62.8±5.7	13.0±5.9	15.5±5.0*	34.3±6.5*
**All Groups**	70.4±0.6	21.2±0.5	28.1±0.5*	21.1±0.6

Table footnote. The phenotypes of at least 50 BrdU+ cells were confirmed using confocal microscopy. Percentages of BrdU+ cells co-expressing the immature neuronal marker doublecortin (DCX) and/or the mature neuronal marker neuronal nucleii (NeuN) are reported. *p<0.05.

To test whether antidepressant treatment impacted the rate of neuronal maturation among BrdU^+^ cells, we next compared the percentages (Table 1) and total numbers (Table 2) of 10–14 Day-old BrdU^+^ cells expressing immature (DCX^+^), transitioning (DCX/NeuN^+^) and mature (NeuN^+^) neuronal phenotypes. In all groups combined, most ∼2 week-old neurons expressed a transitioning versus immature (*p*<0.02) or mature (*p*<0.05) neuronal phenotype (Table 1 ‘All groups’ row; *F*_(2, 42)_ = 3.6; *p*<0.05). Although the total percentage of BrdU^+^ cells expressing neuronal phenotypes was unaffected by treatment (*F*_(4, 21)_ = 2.1; *p*>0.05; Table 1 ‘Neuronal ‘ Column), neuronal maturation stage significantly interacted with antidepressant treatment (*F*_(8, 42)_ = 3.6; *p*<0.01). Specifically, a lower percentage of BrdU^+^ cells co-labeled with DCX/NeuN^+^ (*p<*0.05) and a higher percentage co-labeled with NeuN alone (*p*<0.05) in the dentate gyri of DES-HI-treated rats relative to controls. Similarly, while total new neuron number did not statistically vary across treatment groups (*F*_(4,21)_ = 0.51; *p*>0.05), more mature neurons were detected in the dentate gyri of DES-HI rats versus controls (*p* = 0.003; *F*_(8,42)_ = 3.47; *p*<0.01; Table 2).

**Table 2:** Antidepressant Treatment Impacts Total New Neuron Number.

Group	Neurons	Immature (DCX^+^)	Transitioning (DCX/NeuN^+^)	Mature (NeuN^+^)
**Vehicle**	4434.7±594.1	1243.7±290.4	2000.7±271.9	1190.2±242.4
**Ven-Lo**	4594.3±354.0	1699.3±152.5	2141.8±279.4	752.9±89.0
**Ven-Hi**	4807.1±387.6	1904.7±354.6	1634.1±207.2	1269.0±470.1
**Des-Lo**	4294.3±148.9	922.4±113.4	2055.2±181.1	1316.6±262.1
**Des-Hi**	5269.0±885.9	1024.4±411.2	1195.9±328.8	3048.5±921.6**
**All Groups**	4684.9±225.7	1379.9±144.1	1799.0±125.6	1506.0±254.4

Table footnote. The total number of new BrdU^+^ cells estimated stereologically was multiplied by the % of cells expressing a DCX^+^ immature neuronal, DCX/NeuN^+^ transitioning neuronal or NeuN^+^ mature neuronal phenotype to obtain total new neuron number in the hippocampi of each rat. **p<0.01.

**Figure 4:**
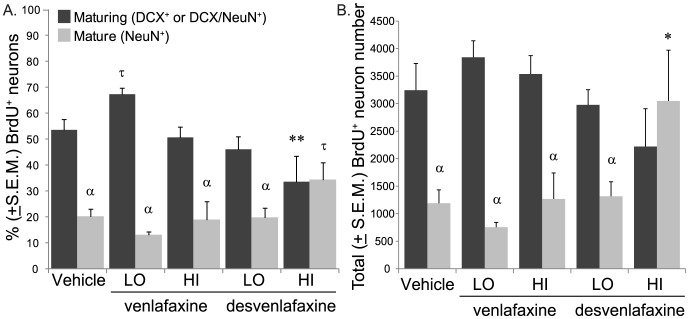
High-dose desvenlafaxine may accelerateneuronal maturation neurons in the denate gyri of adult male rats. **A)** Quantification of maturing (DCX^+^and DCX^+^NeuN^+^)and mature (NeuN^+^ alone) neuronal phenotypesshowed that a significantly higher percentage of BrdU^+^cells expressed maturing versus mature phenotypes in the dentate gyri of vehicle- (p = 0.005), VEN-LO- (p = 0.00008), VEN-HI- (p = 0.009), DES-LO- (p = 0.03) treated rats but similar proportionsexpressed maturing and mature phenotypes in the dentate gyri of DES-HI-treatedrats (*p* = 0.56). Relativeto BrdU^+^ cells in vehicle-treated rats,a significantly lower proportion expressed a maturing neuronal phenotype (**p = 0.005)and more tended to express a mature neuronal phenotype (^τ^p = 0.08). **B)** Quantification of number of new maturing and matureneurons showed that more new maturing versus new mature neurons were foundin the dentate gyri of vehicle- (*p* = 0.03),VEN-LO- (*p* = 0.002),VEN-HI- (*p* = 0.02),DES-LO- (*p* = 0.05)treated rats but similar numbers of maturing and mature new neurons were foundin the dentate gyri of DES-HI-treated rats (*p* = 0.32)and significantly more BrdU^+^ mature neurons werefound in the dentate gyri of DES-HI versus control rats (*p = 0.02).

To further explore the effect of antidepressant treatment on neuron maturation, we compared the percentage (Figure 4A) and total number (Figure 4B) of BrdU^+^ cells expressing maturing (DCX^+^ and DCX/NeuN^+^) versus mature (NeuN^+^) phenotypes. As expected, the percentage of BrdU^+^ cells expressing neuronal phenotypes did not vary by group (*F*_(4, 21)_ = 2.1; *p*>0.05) and as expected of ∼2 week-old BrdU^+^ neurons, more expressed a maturing versus mature phenotype in all rats combined (*F*_(1, 21)_ = 40.5; *p*<0.0001) and phenotype varied significantly by antidepressant treatment group (*F*_(4, 21)_ = 4.7; *p*<0.01). Specifically, significantly more BrdU^+^ cells expressed maturing versus mature phenotypes in the dentate gyri of vehicle- (*p*<0.01), VEN-LO- (*p*<0.0001), VEN-HI- (*p*<0.01), DES-LO- (*p*<0.05) treated rats but similar proportions expressed maturing and mature phenotypes in the dentate gyri of DES-HI-treated rats (*p*>0.05). Relative to BrdU^+^ cells in vehicle-treated rats, a significantly lower proportion expressed a maturing neuronal phenotype (*p*<0.01) and more tended to express a mature neuronal phenotype (0.10>*p>0.05*; Figure 4A). Similarly, total neuron number did not statistically vary by group (*F*_(4, 21)_ = 0.51; *p*>0.05) and although more maturing versus mature neurons were detected in the dentate gyri of all rats combined (*F*_(1, 21)_ = 23.07; *p*<0.0001), this effect interacted with antidepressant treatment (*F*_(4, 21)_ = 3.6; *p*<0.05). More new maturing versus new mature neurons were found in the dentate gyri of vehicle- (*p*<0.05), VEN-LO- (*p*<0.01), VEN-HI- (*p*<0.05), DES-LO- (*p*<0.05) treated rats but similar numbers of maturing and mature new neurons were found in the dentate gyri of DES-HI-treated rats (*p*>0.05) and significantly more BrdU^+^ mature neurons were found in the dentate gyri of DES-HI versus control rats (*p*<0.05; Figure 4B).

### 3.4 Morphological changes undetectable in ∼2 week-old DCX^+^ neurons

The observation that high dose desvenlafaxine treatment may accelerate neuronal maturation, based on the reduced proportions of immature but increased proportions mature neurons detected in the dentate gyri of DES-HI versus vehicle-treated rats led us to hypothesize that DCX^+^ cells in the dentate gyri of these rats may exhibit more mature morphologies. Doublecortin expression begins during the initial steps of neuronal differentiation in unpolarized cells with processes parallel to the granule cell layer and ends when the cells become highly polarized cells with extensively branched processes that can be found in the granule cell and molecular layers [48].

**Figure 5:**
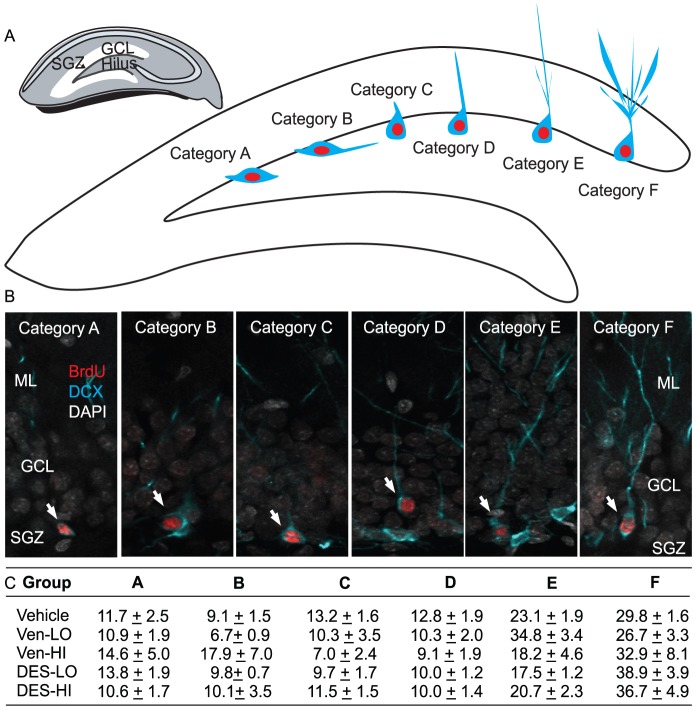
Anti-depressant treatment does not influence the maturation of DCX^+^ neurons found throughout the dentate gyri of adult male rats. **A**) Schematic of the hippocampus proper and dentate gyrus (small cartoon on left) containing the granule cell layer (GCL), subgranular zone (SGZ) where neural progenitor cells divide and hilus. The larger schematic on the right depicts the GCL and hilus of the dentate gyrus with examples of BrdU/DCX^+^ cells classified as Category A-F based upon their dendritic morphologies and extension through the GCL. Note that immature neurons found throughout the infra- and supra-pyramidal blades of the granule cell layer were categorized. This categorization has been used previously to estimate the maturity of DCX^+^ neurons. **B**) Representative confocal images of 10 to 14 day-old BrdU^+^ cells (in red) that express DCX (in cyan) and that have been classified as Category A-F based upon their dendritic morphology and extension through the GCL. Cell nuclei are labeled with DAPI (in gray) and grouped into 6 categories (A to F) with all cell nuclei visualized using DAPI (gray) and the GCL can be visualized in each panel. **C**) Regardless of treatment group, the majority of 10 to 14 day-old BrdU/DCX+ neurons were classified as Category E or F and treatment did not impact Categorization.

Figure 5 shows representative examples of new BrdU/DCX^+^ cells categorized morphologically as A (the most immature unpolarized DCX^+^ neurons with no branches) through F (the most mature highly polarized DCX+ neurons with extensive branching that extended into the GCL and molecular layer) based on the scheme of Kempermann and colleagues [48]. Figure 5C shows that although the percentage of cells categorized as A, B, C, D, E or F statistically differed (*F*_(5,105)_ = 2.98, *p*<0.001), maturation stage neither varied with (*F*_(4,21)_ = 2.25, *p*>0.05) nor interacted with treatment (F_(20, 1095)_ = 1.41, *p*>0.05). Regardless of treatment, significantly more cells were categorized as E (∼22.7%) or F (∼33.1%) versus A (∼12.4%), B (∼11.0%), C (∼10.2%) or D (∼10.4%; all *p values*≤0.0001). More elaborate characterization using high resolution confocal projections of 10 randomly selected Category E and F BrdU/DCX^+^ neurons from each rat confirmed that soma sizes and dendritic branch lengths and numbers were similar across treatment groups (Table 3). These data suggest that any morphological changes that accompany the effects of desvenlafaxine treatment are likely observable in DCX^+^ cells younger than the population of 10–14 day-old BrdU/DCX+ neurons characterized in the current study.

**Table 3:** Morphometric Analysis of BrdU/DCX+ Neurons.

Group	Cell Body Perimeter (mm)	Cell Body Area (mm^2^)	# of Branches	# of Nodes	Total Branch Length/Cell (mm)	Avg Branch Length/Cell (mm)
**Vehicle**	24.3±0.6	46.1±2.4	3.5±0.3	2.3±0.2	275.3±22.3	81.5±3.0
Ven-Lo	23.9±0.3	45.6±1.8	2.9±0.2	1.8±0.1	213.6±15.5	78.1±6.4
Ven-Hi	23.0±0.6	42.7±1.8	3.1±0.2	1.9±0.2	240.9±21.1	79.8±2.4
Des-Lo	24.5±0.4	47.3±2.3	3.3±0.1	1.9±0.2	275.3±20.3	84.7±3.5
**Des-Hi**	24.5±0.5	48.7±1.6	3.6±0.2	2.1±0.1	282.5±25.3	79.6±5.3

## Discussion

Together our data expand the picture of how antidepressants affect adult hippocampal neurogenesis. In agreement with previous work testing the effects of members of other antidepressant classes on adult hippocampal neurogenesis, the high SNRI desvenlafaxine dose used in the current study increased the number of new cells in the hippocampi of adult male rats, but only after 3 days of treatment instead of after several weeks of treatment in the case of other antidepressant drugs. Consistent with the results of previous studies employing SNRIs or members of other antidepressant drug classes, neither SNRI used in the current study impacted the total number of neurons or glia produced, but more new neurons in the dentate gyri of high dose desvenlafaxine-treated rats exhibited mature versus maturing phenotypes. The finding that the high desvenlafaxine dose used in the current study may accelerate neuronal maturation is important because it suggests a potentially novel target for antidepressant strategy development.

A high-dose desvenlafaxine course initiated 3 days before BrdU labeling increased new cell number in the adult rat dentate gyrus (Figure 2B). In contrast, several weeks-long but not acute SSRI fluoxetine (5–20 mg/kg), MAOI tranylcypromine (10 mg/kg), TCA imipramine (10 mg/kg), or NRI reboxetine (20 mg/kg) treatments before BrdU labeling were reported to increase new cell number by stimulating NPC proliferation [11], [12], [24], [49]. Interestingly, a 2 week-long 10 mg/kg intraperitoneal subcutaneous venlafaxine treatment stimulated NPC proliferation in previous studies but a 2 week-long 40 mg/kg course did not [11], [14]. Whether the latter dose, ∼8 times the current maximum recommended clinical venlafaxine dose [39], counteracted the beneficial effect of the lower dose on neurogenesis through a side effect is unclear. In addition, injection stress can impact neurogenesis [50], and repeated subcutaneous injections could be more stressful than repeated intraperitoneal injections. We suspect that longer duration venlafaxine and low-dose desvenlafaxine courses before BrdU labeling would increase new cell number, consistent with reports using venlafaxine or other antidepressant classes [11], [12], [49]. In fact, Figure 2 shows that although dose-dependent effects of venlafaxine and desvenlafaxine appear to emerge, they do not achieve statistical significance. Nonetheless, the high desvenlafaxine dose used in the current study impacted neurogenesis more rapidly than has been reported for other antidepressants including venlafaxine [11], [12], [14], [49].

Because we employed a ∼2 week survival period after BrdU injection, the high desvenlafaxine dose used in the current study could have also increased total new cell number by promoting new cell survival. A few studies have specifically tested the effect of antidepressants on new cell survival by initiating antidepressant treatment the day after BrdU labeling. Using this approach, Malberg and colleagues [12] found that a 2 week-long 5 mg/day fluoxetine treatment did not potentiate the survival of new cells but Wang and colleagues [15] found that a 4 week-long 18 mg/day fluoxetine treatment did potentiate new cell survival. Khawaja and colleagues [11] found that although chronic venlafaxine and fluoxetine treatment potentiated NPC proliferation similarly and venlafaxine potentiated new cell number to a greater extent than fluoxetine when the survival period was extended by 4 weeks, suggesting that venlafaxine promoted new cell survival. In the current study, more 10-to-14 day-old cells were detected in the dentate gyri of DES-HI treated rats (Figure 2B). Future experiments specifically testing the effects of desvenlafaxine on NPC proliferation versus survival would provide more insight about the mechanisms by which an acute course of this SNRI increases the total new cell number. Although more new cells were found in the dentate gyri of DES-HI-treated rats, consistent percentages of BrdU^+^ cells expressed neuronal and glial phenotypes across treatment groups suggesting that the fate choice of new cells was unaffected by the antidepressants used in the current study (Figure 3). As expected of 10–14 day-old BrdU^+^ cells in the hippocampus of adult rats, the majority expressed neuronal phenotypes and fewer than 5% expressed NG2^+^ oligodendrocyte precursor or GFAP^+^ astrocyte phenotypes [4], [5], [43], [47], [51], [52]. Approximately 20% of the BrdU^+^ cells did not co-label with the phenotypic markers employed in the current study. These cells could include quiescent GFAP^−^ progenitor cells, mature oligodendrocytes and S100β^+^/GFAP^−^ astrocytes [53]. Our data are consistent with previous work showing that antidepressants potentiate NPC proliferation and perhaps new cell survival but do not impact the fate choice of new cells in the adult rodent hippocampus.

However, the most exciting finding of our study emerged when we focused specifically upon characterizing new neurons. We found that a smaller percentage of 10–14 day-old BrdU^+^ neurons expressed maturing DCX^+^ or DCX/NeuN^+^ phenotypes and a larger percentage expressed a mature NeuN^+^/DCX^−^ phenotype in the dentate gyri of high desvenlafaxine dose-treated rats versus controls (Table 1 and Figure 4). These rats had significantly more mature neurons than control rats (Table 2 and Figure 4). DCX is a microtubule-associated protein expressed by migrating neuronal precursor cells and immature neurons in the embryonic and adult CNS that has recently been shown to regulate dendritic and axonal outgrowth [54]. In the adult rodent hippocampus, new neurons begin to down-regulate doublecortin expression after about 2 weeks when they begin expressing the mature neuronal protein neuronal nuclei (NeuN); few new neurons retain DCX expression after approximately one month [47], [51], [55]. NeuN is expressed in the nuclei and cytoplasm of most mature neurons and has recently been identified as the Fox-3 gene product that functions as an mRNA splicing regulator, potentially associated with the production of synaptic proteins [56]. Based upon our understanding about the functional role of the proteins used as markers of neuronal maturation in the current study, the 20 mg/day desvenlafaxine dose appears to have accelerated the maturation of new neurons through the migration and process extension phases. Importantly, future work should test whether similar effects on neurogenesis that are related to the reversal of behavioral symptoms are observed in animal models of depression.

Accelerated neuronal maturation has also been reported to occur in the hippocampi of mice, but only after chronic, but not acute, fluoxetine treatment [15]. In mice, chronic fluoxetine treatment increase dendritic length and branching complexity in 2 day-old and 4 week-old DCX^+^ hippocampal neurons suggesting that these neurons were more rapidly integrated functionally than those in control mice [15]. We did not detect similar desvenlafaxine-induced morphological changes in ∼2 week-old BrdU/DCX^+^ neurons in the hippocampi of rats in the current study (Figure 5 and Table 3). A recent report has shown that ECS alters the morphology but not number of dendritic spines on new neurons in the adult rat hippocampus [57], suggesting that overt antidepressant-induced morphological changes in new neurons may be less robust in the adult rat versus mouse hippocampus. In addition, we may have detected these changes by looking at younger than ∼2 week-old BrdU/DCX^+^ neurons. Interestingly, in the adult hippocampus, younger (<4 weeks-old) neurons exhibit reduced LTP induction thresholds and small LTP amplitudes whereas 1–1.5 month old neurons exhibit lower LTP induction thresholds and larger NR_2_B-containing NMDA receptor-dependent LTP amplitudes [58]–[61] before becoming electrophysiologically indistinguishable from mature granule neurons months later [28]. Future work exploring the relationship between markers of morphological and electrophysiological maturity should provide insight into how neurogenesis is impacted by antidepressants functionally.

Most antidepressants increase extracellular 5-HT and/or NE levels [1] and these neurotransmitters impact adult hippocampal neurogenesis [12], [19], [62], [63]. SNRI antidepressants increase levels of these neurotransmitters variably [64]. Venlafaxine and desvenlafaxine preferentially antagonize 5-HT transporter proteins at all doses and NE transporter proteins at higher doses [35]–[37], [64], suggesting that the high desvenlafaxine dose used in the current study may have rapidly increased hippocampal neurogenesis through dual effects on 5-HT and NE levels not achieved by the lower dose. In addition, desvenlafaxine exhibits a slightly higher affinity for the 5-HT transporter protein and a much higher affinity for the NE protein and therefore, could have produced its effects at the higher dose through a NE-linked mechanism not activated by the same dose of venlafaxine. Our finding that the high desvenlafaxine but not venlafaxine dose affected neurogenesis is also consistent with published data showing that desvenlafaxine produces faster and higher steady state plasma and brain *O*-desmethylvenlafaxine concentrations with slower clearance rates than the same clinical dose of venlafaxine [39]–. Thus, a higher venlafaxine dose could produce the same rapid effects on adult hippocampal neurogenesis that the 20 mg/kg dose of desvenlafaxine produced in the current study either directly or because higher levels of its primary active metabolite O-desmethylvenlafaxine would be achieved. Since ∼29% and ∼26% of venlafaxine is excreted as unconjugated and conjugated O-desmethylvenlafaxine, respectively, in subjects considered to be extensive cytochrome P450 enzyme CYP2D6 metabolizers, the dose would likely need to be increased 2- to 3-fold to work through this mechanism. Note that a 40 mg/kg venlafaxine dose did not impact neurogenesis in a previously published study [14]. Our data do suggest that future work comparing the rapidity with which variable doses of SNRIs that differentially impact 5-HT/NE level ratios impact adult neurogenesis may provide insight for the development of novel antidepressant drug strategies.

SNRI antidepressants may also impact adult neurogenesis through mechanisms exploited by members of other antidepressant classes that more selectively modulate 5-HT or NE neurotransmission. For example, elevated NE levels may stimulate the proliferation of more naïve and normally quiescent Type 1 GFAP^+^ hippocampal progenitor cells through β_3_-adrenergic receptor activation [62], [63]. In addition, antidepressants that affect serotonin neurotransmission typically activate postsynaptic hippocampal 5-HT_1A_ receptors and desensitize raphe 5-HT_1A_ autoreceptors to induce a more generalized 5-HT level increase [65]. Acute or chronic hippocampal 5-HT_1A_ receptor activation can stimulate NPC proliferation and promote the survival of new cells [66]. Antidepressants can also increase the expression of neurotrophic factors that include brain-derived neurotrophic factor, vascular endothelial growth factor and nerve growth factor and their receptors that include TrkB and Flk1, which have all been shown to stimulate adult neurogenesis [1], [33], [52], [67]–[70]. The effects of stress and HPA activation on hippocampal neurogenesis (for review see [18]) and serotonergic transmission are well-documented and antidepressants can impact HPA axis activity and limbic levels of adrenocorticotropin hormone and corticosterone (for review see [69], [71]). Of course, desvenlafaxine succinate could have impacted hippocampal neurogenesis more rapidly in the current study than other SNRIs and members of other antidepressant classes by activating a wider variety of neurogenic mechanisms that will be revealed as research on these newer drugs progresses. Future work examining whether proportions of maturing and mature neurons differ at variable survival times and whether younger than 2 week-old DCX^+^ neurons exhibit morphological maturation in antidepressant versus control rats would corroborate our interesting effects.

## Conclusions

Our results show that the SNRI antidepressant desvenlafaxine succinate, but not venlafaxine potentiates adult hippocampal neurogenesis by enhancing the production and potentially survival of new cells, but works far rapidly than members of other antidepressant classes that mediate similar effects after several weeks. The most important findings of the study were that desvenlafaxine succinate did not alter the proportion of new granule neurons generated but did accelerate their rate of maturation. The pharmacokinetics of desvenlafaxine succinate versus other antidepressants, including venlafaxine, implies a faster response onset that may be linked to its rapid effects on adult neurogenesis that we found in the current study. Future work exploring whether rapid effects of putatively fast acting antidepressants on neurogenesis relate to a more rapid alleviation of depressive symptoms in animal models of depression will be critical for understanding the role that neurogenesis plays in mood disorders and recovery from mood disorders.
